# Targeting IL-11 signaling in colon cancer

**DOI:** 10.18632/oncotarget.1410

**Published:** 2013-10-25

**Authors:** Matthias Ernst, Tracy L. Putoczki

**Affiliations:** The Walter and Eliza Hall Institute for Medical Research and Department of Medical Biology, University of Melbourne, Victoria, Australia; The Walter and Eliza Hall Institute for Medical Research and Department of Medical Biology, University of Melbourne, Victoria, Australia

**Figure 1:**
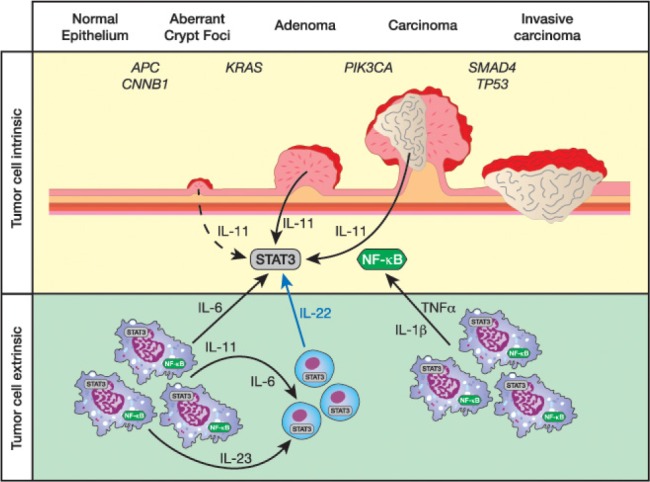
Cytokines in the tumor microenvironment promote colorectal cancer progression Although colorectal cancers arise from a step-wise accumulation of mutations in oncogenes and tumor suppressors (top half), the growth and survival of the neoplastic epithelium is modulated by cytokine-mediated activation of the latent transcription factors NF-κB and Stat3. Many of these cytokines are produced by myeloid (purple) and lymphoid cells subsets (blue) that accumulated in the tumor microenvironment (bottom half). Some of the Stat3 activating cytokines that promote (colitis-associated) colon cancer in mouse models are indicated. Note, that the tumor epithelium can produce IL-11 as part of an auto-/paracrine feed forward loop [4,6]

The development of Colorectal cancer (CRC), the fourth most common cause of malignancy worldwide, is driven by the sequential accumulation of genetic changes. In the Western world, more than 80% of sporadic CRC cases arise from aberrant activation of the WNT-pathway, including mutations in the *adenomatous polyposis coli (APC)* tumor suppressor gene and *CTNNB1* transcription factor, and followed by mutations in *K-RAS, PIK3CA, SMAD4* and *TP53* as the tumor emerges and progresses [1] (Fig 1, top). Recent evidence suggests that these tumor intrinsic events are accompanied by ‘inflammatory gene signatures’ [2], which were originally identified in colitis-associated cancer (CAC) and have long been suspected to account for the increased CRC lifetime risk in patients suffering from ulcerative colitis (UC). Inflammation associated with UC is characterized by myeloid cells and T-helper interleukin (IL)-17-producing (Th17) cells, which promote tumorigenesis and are associated with decreased disease-free CRC patient survival, rather than the CD4/CD8 T-cells that constitute a positive prognostic sign in these patients [3].

With the advent of cell-type specific gene inactivation in mice, it became possible to dissect the molecular mechanisms by which the inflamed tumor microenvironment increased the tumor cells' capacity to survive and proliferate. For instance, myeloid and epithelial cell-specific ablation of NF-κB signaling reduced tumor development in CAC-challenged mice [4]. This implied a role for myeloid cell-derived cytokines produced by these sentinels in response to engagement of pathogen recognition receptors. Therapeutic benefits therefore arose in these models in response to suppression of TNFα, IL-1β, IL6 or IL-23, whereby the later cytokine is likely to promote tumor growth indirectly by enabling a tumorigenic Th17-cell response [5] (Fig 1, bottom). Akin to NF-κB signaling, epithelial ablation of Stat3, a transcription factor engaged by cytokines of the IL-6/-11, IL-10/-22, and IL-12/-23 cytokine families, also reduced CAC-associated tumor burden [4]. However, myeloid-cell specific Stat3 ablation triggered the development of enterocolitis, consistent with its identification as a UC susceptibility locus and molecularly reconciled with the failure for IL-10 to dampen the host's ensuing immune response. Emerging evidence firmly cements an important role for epithelial Stat3 not only for homeostatic turnover of the intestinal epithelium, but also to ensure its efficient recovery from the continuous mechanical trauma and exposure to a noxious environment. Absence of IL-22 or its sequestration from the IL-22 receptor therefore increases susceptibility to colitis. Likewise, mice harboring mutations in the shared IL-6/-11 cytokines family receptor gp130 that impair Stat3 activation are prone to ulceration, while gp130 mutations in mice with a converse effect confer resistance to colitis [4]. Given the molecular similarities between wound healing and tumor promotion, it is therefore not surprising that the latter cytokines have all been functionally implicated in enhancing CAC.

Although IL-6 has been associated with many epithelial cancers, including those of the gastrointestinal tract, where elevated serum IL-6 often serves as an indicator of poor prognosis, we have recently found that IL-11 acts as a more potent driver for CAC and indeed also in mouse models of sporadic CRC [6]. Analysis of bone-marrow chimeras incapable of responding to IL-11 suggests that IL-11 directly activates the neoplastic epithelium rather than promoting tumorigenesis indirectly by engaging hematopoietic cells. The latter finding is surprising in light of the clinical use of IL-11 to boost platelet production, but is consistent with IL-11's ability to stimulate CRC cell lines and its proposed clinical use to prevent the acute mucositis associated with cytotoxic cancer therapy [7]. At first sight, the dichotomy in epithelial response to IL-6 and IL-11 is surprising given the shared use of the gp130 receptor and the highly overlapping gene expression signatures elicited by the two cytokines. It is, however, likely that expression of the IL-11 specific co-receptor, but not its IL-6 specific counterpart, is enriched in cells of the epithelial stem and progenitor compartment [6]. This hardwiring arrangement may uncouple a (undesired) mucosal response from the widespread IL-6 expression associated with systemic inflammation, while still allowing cells to respond to local inflammation at the site of mucosal injury.

In the aforementioned mouse models of CAC, the maintenance of established tumors is absolutely dependent on continuous IL-11-dependent Stat3 activation. Likewise, the absence of IL-11 signaling in models of sporadic CRC correlates with growth arrest of the neoplastic epithelium that harbors activating mutations of the WNT-pathway (including APC and CTNBB1), thereby implying a rate limiting function for IL-11/gp130/Stat3 signaling in potentially a majority of CRCs [6]. Since epithelial Stat3 activity in human CRC is often pronounced at the invasive tumor front, and since CRC stem-like cells more readily respond to IL-11 than IL-6, anti-IL-11 therapy may interfere with clinically relevant facets of these cancers. Thus targeting IL-11 signaling fits the emerging concept of combating tumor progression through inhibition of growth promoting cytokines, their receptors and associated signaling pathways. Clinical trials with these emerging anti-cytokine drugs, either as mono or adjuvant therapy, will identify the most suitable subsets of CRC patients for specific treatment combinations.
